# FluBreaks: Early Epidemic Detection from Google Flu Trends

**DOI:** 10.2196/jmir.2102

**Published:** 2012-10-04

**Authors:** Fahad Pervaiz, Mansoor Pervaiz, Nabeel Abdur Rehman, Umar Saif

**Affiliations:** ^1^School of Science and EngineeringComputer Science DepartmentLahore University of Management SciencesLahorePakistan

**Keywords:** Influenza, public health, epidemics, statistical distributions, early response

## Abstract

**Background:**

The Google Flu Trends service was launched in 2008 to track changes in the volume of online search queries related to flu-like symptoms. Over the last few years, the trend data produced by this service has shown a consistent relationship with the actual number of flu reports collected by the US Centers for Disease Control and Prevention (CDC), often identifying increases in flu cases weeks in advance of CDC records. However, contrary to popular belief, Google Flu Trends is not an early epidemic detection system. Instead, it is designed as a baseline indicator of the trend, or changes, in the number of disease cases.

**Objective:**

To evaluate whether these trends can be used as a basis for an early warning system for epidemics.

**Methods:**

We present the first detailed algorithmic analysis of how Google Flu Trends can be used as a basis for building a fully automated system for early warning of epidemics in advance of methods used by the CDC. Based on our work, we present a novel early epidemic detection system, called FluBreaks (dritte.org/flubreaks), based on Google Flu Trends data. We compared the accuracy and practicality of three types of algorithms: normal distribution algorithms, Poisson distribution algorithms, and negative binomial distribution algorithms. We explored the relative merits of these methods, and related our findings to changes in Internet penetration and population size for the regions in Google Flu Trends providing data.

**Results:**

Across our performance metrics of percentage true-positives (RTP), percentage false-positives (RFP), percentage overlap (OT), and percentage early alarms (EA), Poisson- and negative binomial-based algorithms performed better in all except RFP. Poisson-based algorithms had average values of 99%, 28%, 71%, and 76% for RTP, RFP, OT, and EA, respectively, whereas negative binomial-based algorithms had average values of 97.8%, 17.8%, 60%, and 55% for RTP, RFP, OT, and EA, respectively. Moreover, the EA was also affected by the region’s population size. Regions with larger populations (regions 4 and 6) had higher values of EA than region 10 (which had the smallest population) for negative binomial- and Poisson-based algorithms. The difference was 12.5% and 13.5% on average in negative binomial- and Poisson-based algorithms, respectively.

**Conclusions:**

We present the first detailed comparative analysis of popular early epidemic detection algorithms on Google Flu Trends data. We note that realizing this opportunity requires moving beyond the cumulative sum and historical limits method-based normal distribution approaches, traditionally employed by the CDC, to negative binomial- and Poisson-based algorithms to deal with potentially noisy search query data from regions with varying population and Internet penetrations. Based on our work, we have developed FluBreaks, an early warning system for flu epidemics using Google Flu Trends.

## Introduction

Infodemiology introduced the use of nontraditional data sources for the detection of disease trends and outbreaks [1]. These data sources include search queries, social media, Web articles, and blogs posts, which are now being used for real-time disease surveillance [1-4]. In terms of search queries as a source, interest in using these to predict epidemics has been growing recently [5-8]. Most notably, the Google Flu Trends [9] service was launched in 2008 as a way to track changes in the volume of online search queries related to flu-like symptoms [5]. Google Flu Trends provides search query trend data that are real-time and reported on a daily basis, and have been shown to predict the actual cases of a disease such as flu at least 2 weeks ahead of the US Centers for Disease Control and Prevention (CDC).

In the absence of other real-time disease surveillance mechanisms, services such as Google Flu Trends are vitally important for the early detection of epidemics. Existing research on using Google Flu Trends for epidemic detection has focused on addressing this need by collecting data related to the volume of queries for disease symptoms. This work demonstrates that Google search query trends closely follow the actual disease cases reported by the CDC. While these results provide strong support for the potential use of Google Flu Trends data as a basis for an early warning system for epidemics, existing research needs to be advanced along two essential directions to realize this opportunity. First, there is a need to rigorously explore and evolve algorithms for higher-level inference from the Google Flu Trends data that can generate alerts at early stages of epidemics. In particular, the ability of existing approaches to collect raw search volume data needs to be supplemented with computational intelligence to translate these data into actionable information. Second, there is also a need to develop a more detailed appreciation of how changes in population size and Internet penetration affect the ability of a system based on Google Flu Trends data to provide accurate and actionable information.

In this study, we aimed to provide new insights related to these opportunities. We built upon Google Flu Trends data and compared the accuracy and practicality of widely used algorithms for early epidemic detection. These algorithms are classified into three categories based on the type of data distribution they expect. The classifications in question are normal distribution algorithms, Poisson distribution algorithms, and negative binomial distribution algorithms. For normal distribution algorithms, we used cumulative sum (CUSUM) [10-12], the historical limits method (HLM) [10,13], and historical CUSUM (HCusum) [14,15]. For Poisson distribution algorithms, we used Poisson outbreak detection (POD) [16], SaTScan [17], and Poisson CUSUM (PSC) [18,19]. For negative binomial distribution algorithms, we used negative binomial CUSUM (NBC) [20,21] and historical NBC. Some of these algorithms have already been compared on Ross River disease data [22]. Our choice of some of the algorithms (CUSUM, HLM, POD, SaTScan, and NBC) and parameters was also based on this work [22]. However, our work performed the comparison on Google Flu Trends data. We quantitatively compared the accuracy, specificity, and sensitivity of these algorithms, as well as the impact of leveraging information in baseline training periods, seasonal changes, population sizes, and Internet penetrations, on their suitability for detecting epidemics.

## Methods

### Data Sources

#### Google Flu Trends

Traditional disease surveillance networks such as the CDC take up to 2 weeks to collect, process, and report disease cases registered at health centers [23].

Google Flu Trends [9], on the other hand, provides near real-time data on disease cases by benefiting from the likelihood that many patients with flu symptoms search online for their symptoms and remedies before visiting a doctor.

Google Flu Trends compares the popularity of the 50 million most common Google search queries in the United States with flu-like illness rates reported by the CDC’s national surveillance program. The Flu Trends data are derived from a pool of 45 search terms that relate to symptoms, remedies, and complications of flu and generate a trend that closely correlates with CDC data on influenza-like illnesses.

In our experiments, we used Google Flu Trends data from the 9 years between 2003 and 2011.

#### CDC‘s Outpatient Illness Surveillance

Information on patient visits to health care providers in the United States for influenza-like illness is collected through the Outpatient Influenza-like Illness Surveillance Network (ILINet). ILINet consists of more than 3000 health care providers in all 50 states, reporting over 25 million patient visits each year. Each week, approximately 1800 outpatient care sites around the United States report data to the CDC on the total number of patients seen and the number of those patients with influenza-like illnesses. For this system, an influenza-like illness is defined as fever (temperature of 100°F [37.8°C] or greater) and a cough or a sore throat in the absence of a known cause other than influenza. Sites with electronic records use an equivalent definition as determined by state public health authorities. The percentage of patient visits to health care providers for influenza-like illnesses reported each week is weighted on the basis of a state’s population. This percentage is compared each week with the national baseline of 2.5%. The baseline is the mean percentage of patient visits for influenza-like illnesses during noninfluenza weeks for the previous three seasons plus 2 standard deviations [24].

In our experiments, much like Google Flu Trends data, we used CDC influenza-like illness data from the 9 years between 2003 and 2011. Though the CDC has missing data in the nonflu season between 2009 and 2010, we believe this had a minimal effect on our quantitative comparison.

#### Outbreak

For determining periods of outbreaks, the starting points in the time and the duration, we consulted two epidemiologists from different institutes. The first was from the Institute of Public Health, Lahore, Pakistan (responsible for informing the provincial health ministry about disease outbreaks) and the second was from Quaid-e-Azam Medical College, Bahawalpur, Pakistan. These original outbreaks were marked on CDC influenza-like illness data [23].

### Outbreak Detection Algorithms

The early epidemic detection algorithms that we have used are divided into three categories, based on the expected distribution in data: (1) normal distribution algorithms: these expect normal distribution in the data, (2) Poisson distribution algorithms: these expect a Poisson distribution, and (3) negative binomial distribution algorithms: these expect a negative binomial distribution.

#### Normal Distribution Algorithms

The algorithms classified in this category are the Early Aberration Reporting System (EARS) algorithm (CUSUM), HLM, and HCusum.

##### Early Aberration Reporting System Algorithms

**Figure 1:**
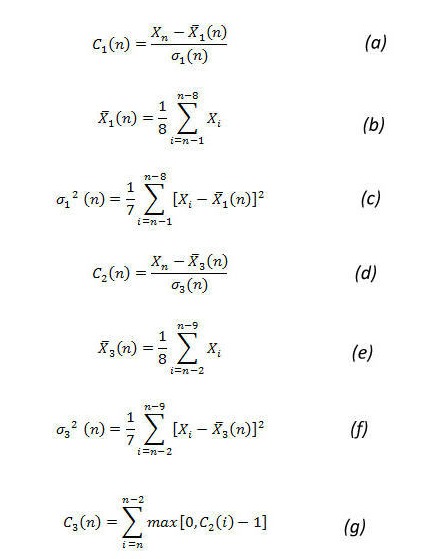
Early Aberration Reporting System (EARS) algorithm equations. C_1_ = cumulative sum (CUSUM) score of C1 algorithm, C_2_ = CUSUM score of C2 algorithm, C_3_ = CUSUM score of C3 algorithm, sigma = standard deviation, X-bar = mean number of cases, X_n_ = number of cases in current time interval. Subscripts refer to a specific variable being linked to either of the three algorithms.

EARS was developed and used by the CDC. EARS comprises three syndromic surveillance early event detection methods called C1, C2, and C3 [11], which are the Shewhart variations of the CUSUM method. These methods use a moving average and standard deviation to standardize the number of occurrences in the historical data. In our analysis, C1 used the 4 weeks prior to the current week of observation for calculating the average and standard deviation. The value of average and standard deviation is used to determine the C1 score (Figure 1, parts a, b, and c). C2 is similar to C1 but used the 4 weeks after a 1-week lag. It means that it used week 2 to week 5 for calculating the average and standard deviation (Figure 1, parts d, e, and f). C3 used the C2 score for the previous 3 weeks to calculate the C3 score, as shown in Figure 1 (part g).

The C1, C2, and C3 EARS algorithms require a baseline (training period) and cut-off (threshold) as parameters. In our experiments, we used both 4 weeks and 8 weeks as the baseline. A shorter training period (baseline) has been shown to insulate CUSUM from seasonal changes [15]. For each of these baseline periods, we compared the algorithms for four cut-off values: 2, 4, 6, and 8. This means that we declared an outbreak if the observed value exceeded the mean value more than a standard deviation of 3, 5, 7, and 9, respectively. A higher cut-off point makes the algorithm less responsive to transient changes in the rate of occurrence of disease cases. In our analysis, we excluded C1 with cut-offs of 6 and 8 for both baselines because it rarely raised an outbreak alarm for the 9 years of data.

Since CUSUM uses mean and standard deviation for raising alarms, it is best for outbreaks with respect to normal distribution of data. It means that the algorithm is very sensitive to a sudden rise, which generates an early alarm. In addition, it expects a constant rise in data for an outbreak to continue because the start of a rise becomes part of historical data, consequently also raising the mean and standard deviation for the algorithm.

##### Historical Limits Method

**Figure 2:**
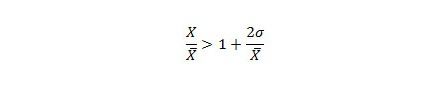
Historical limits method (HLM) equation. Sigma = standard deviation, X = number of reported cases in the current period, X-bar = mean.

The CUSUM methods used in EARS do not account for seasonality by design; however, the HLM incorporates historical data. In HLM an outbreak is signaled when the identity in Figure 2 is true.

**Figure 3:**
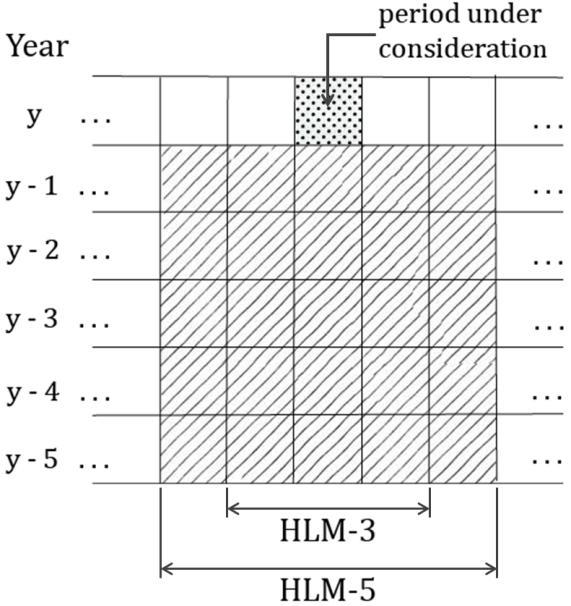
Historical data of the historical limits methods (HLM). HLM-3 = 3 consecutive weeks in the historical data, HLM-5 = 5 consecutive weeks in the historical data.

In the HLM, the system determines the expected value of a week by (1) using 3 consecutive weeks for every year in the historical data, which is the current week, the preceding week, and the subsequent week (entitled HLM-3), and (2) using 5 consecutive weeks for every year, in the past years: the current week, the preceding 2 weeks, and the subsequent 2 weeks (HLM-5) in the historical data (Figure 3).

The above two variations (HLM-3, which uses 15 baseline points, and HLM-5, which uses 25 baseline points) are recommended by Pelecanos et al [22].

We used both HLM-3 and HLM-5, in which the training period comprised 5 years, starting from 2003 and ending in 2008. For determining outbreaks within the training period, we removed 1 year at a time from the timeline between 2003 and 2008 (both years inclusive). Then we assumed that the remaining years were consecutive and determined outbreaks during the omitted year by using the remaining 4 years. This process was repeated for every year of the training period.

Just like EARS, HLM runs on the mean and standard deviation of the data. Therefore, the definition of outbreak is to expect a normal distribution and to mark any outlier, according to normal distribution, as an outbreak.

##### Historical CUSUM

**Figure 4:**
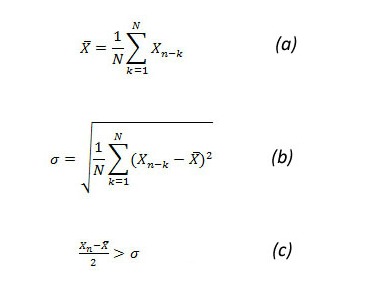
Historical cumulative sum (HCUSUM) equations. Sigma = standard deviation, X_n_ = number of cases in current time interval, X-bar = mean number of cases. N = 5, as the baseline period is the preceding 5 years.

HCusum is a seasonally adjusted CUSUM [15]. It considers the same period during previous years before creating an alert. This ignores the regular trend of rises in count data and signals only when an anomaly happens. Hence, the baseline data of our calculation was the patient count for the same week number during the preceding 5 years. The mean (X-bar) gives a reference value of what an expected count will be. Sigma gives an insight into how much variation there has been in the values used to calculate the expected value [14] (Figure 4, parts a and b).

An outbreak is declared if the identity in (c) is true.

#### Poisson Distribution Algorithms

The algorithms classified in this category are POD, SaTScan, and PSC.

##### Poisson Outbreak Detection Method

The POD method assumes that the number of cases follow a Poisson distribution. The POD method [16] uses 10 years of historical data for calculating the incidence rate of a disease. This 10-year period is used for accommodating high variability and skewed distribution of the seasonal incidence rates. To accommodate variability in the population of the various regions, if the population of a certain region is less than 2500, the crude incidence rate is used for determining outbreaks. If the population size is larger than 2500, then the crude incidence rate is used if either the maximum number of notifications is less than 5 or the crude incidence rate and trimmed incidence rate differ by less than 20%. (Trimmed incidence rate is calculated by omitting the years with the maximum or minimum number of cases). Otherwise, the median incidence rate is used. An outbreak is considered if the chance of the actual number of cases occurring was less than 1%. For POD, a year is divided into seasons (winter, spring, summer, and autumn) and the IRs are calculated for each season rather than the whole year. This is how POD caters for seasonality. Since it is Poisson-based algorithm, it is the best fit when the outbreak data’s variance to mean ratio (VMR) equals 1. This value of VMR implies that the data follow a Poisson distribution.

We followed certain suggestions from Pelecanos et al [22] and increased the percentage chance from 1% to 5%. This is because we did not have 10 years’ worth of historical data to train the system. Therefore, this change in percentage helped reduce the sensitivity of the algorithm. We used the first 5 years as a training period and then added every subsequent year for further outbreak detection.

##### Purely Temporal SaTScan

The SaTScan algorithm can be used for spatial analysis, temporal analysis, and spatiotemporal analysis. We used only the temporal analysis for our outbreak detection, since spatial mapping is already fixed to a CDC-defined region. We used a Poisson permutation, which works best for data following a Poisson distribution. This is the case when the data’s VMR is equal to 1.

Temporal SaTScan creates 1-dimensional clusters by sliding and scaling a window within an interval of 60 days. We relied on the Poisson permutation to determine the clusters with the highest likelihood ratio.

**Figure 5:**
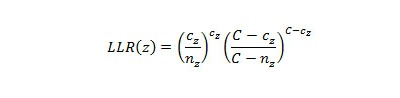
SaTScan equation. C = total number of cases, C_z_ = observed number of cases in window z, LLR = likelihood ratio, n_z_ = expected number of cases or population in window z.

The equation (Figure 5) calculates the log likelihood ratio for the selection of the cluster.

Once we had the best cluster within an interval, the algorithm calculated the *P *value of the cluster using Monte Carlo testing. A *P *value less than .001 determines, with high significance, that there is an outbreak in the cluster.

SaTScan does not accommodate seasonality. Therefore, to adjust SaTScan for seasonality we scaled the population size of the region under analysis on a weekly basis. SaTScan uses population size as one of the parameters, so for every week the population is scaled. The factor for scaling the population size is dependent on the incidence rate for each week and the annual population: *population* *i* = annual population * (incidence rate for week i / total incidence rate), where *population* *i *is the scaled population for a particular week, *annual population *is the population of the year under observation, the *incidence rate for week i *is the average incidence rate of a particular week over the past years, and the *total incidence rate *is the average of incidence rates for all weeks through the year.

Moreover, as CDC and Google data are reported on a weekly cycle, we parameterized SaTScan on a weekly time unit. We set the *P *value cut-off at .001 (to avoid detecting smaller clusters in response to seasonal changes) and set the number of iterations to 15 (since our data comprised 8 flu seasons). To detect a new cluster in each iteration, we set the iterative scan to adjust for more likely clusters. We did not change the default value of the maximum Monte Carlo replication (999).

##### Poisson CUSUM

**Figure 6:**
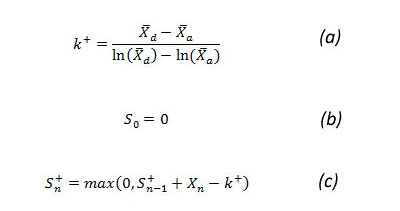
Poisson cumulative sum (CUSUM) equations. k = reference value, S_n_ = CUSUM score, X_n_ = number of cases in current time interval, X-bar_a_ = null hypotheses mean, X-bar_d_ = alternative hypotheses mean, + superscript refers to values always being positive.

PSC is an algorithm that detects the anomalies efficiently in data that follow a Poisson distribution [18,19]. It tests a null hypothesis that the current value is in control and the alternative hypothesis that the value is out of control. As a Poisson distribution can be defined by only one parameter (mean), the reference values for both hypotheses are taken as the mean. The reference for the null hypothesis is the average value (X_a_-bar) of the data in the baseline window. The baseline window is the period of the past 7 weeks from the current week of analysis, with a 1-week guard band in between. For the alternative hypothesis, the mean value (X_d_-bar) is the sum of the average and 2 times the standard deviation of the baseline period. (X_a_-bar) and (X_d_-bar) are used in calculating *k* ^+ ^of the PSC, as shown in Figure 6 (part a), which also shows the equations used to calculate CUSUM (parts b and c).

An outbreak is signaled when the computed CUSUM score is higher than the threshold *h*. The threshold *h *is equal to t * k [19]. We did our analysis with *t *= 1 and *t *= 1.5.

#### Negative Binomial Distribution Algorithms

This category comprises NBC and historical NBC.

##### Negative Binomial CUSUM

###### Static Thresholds

**Figure 7:**
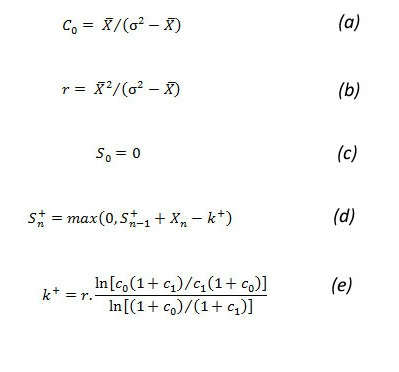
Negative binomial cumulative sum (CUSUM) equations. k = reference value, (r,c) = parameters of negative binomial distribution, S_n_ = CUSUM score, sigma =standard deviation, X_n_ = number of cases in current time interval, X-bar = mean number of cases, + superscript refers to values always being positive.

We selected NBC [20,21] because of its property of handling inaccuracies due to overdispersion in data. Overdispersion in data causes VMR to be higher than 1. This generally happens during seasonal increase. Two parameters, (*r*) and (*c_0_*), are used to describe negative binomial distribution. Equations in Figure 7 (parts a and b) are used to determine the value of these parameters based on mean (X-bar) and variance (sigma^2^), which are derived from the baseline period. The decision interval is given by equations in Figure 7 (parts c, d, and e) through looking for changes in c from an in-control c_0 _to an out-of-control c_1_, where c_1 _> c_0 _[20].

The out-of-control level c_1 _is determined by adding 2 times the standard deviation of the baseline period to the mean of the baseline. We kept a baseline interval of 7 weeks and a guard band of 1 week. The guard band prevents the most recent data from being included in baseline calculations. Therefore, the baseline period and current week will have a gap of 1 week as a guard band. The CUSUM score is compared with the threshold value *h*. An outbreak is declared if the CUSUM score (S_n_^+^) > *h. *The results were calculated using static cut-off (threshold) values of 8 and 15 [22].

###### Variable Thresholds

NBC with static threshold, although it catches the longevity of the outbreak, is sensitive in raising early alarms. To cater for this sensitivity we introduce variable thresholds for NBC. A new parameter, *h* *v*, is calculated, which is used as the threshold for the CUSUM score. The calculation of the rest of the parameters is based on equations in Figure 7. The variable threshold *h* *v *is calculated by the equation *h* *v* = t * k, where *t *is a constant. We performed our analysis with values of *t *of both 1 and 1.5. Involving *k *in the threshold calculation changes the cut-off values with the variation in the count data of the baseline window. This reduces the sensitivity of the CUSUM [19].

##### Historical Negative Binomial CUSUM

**Figure 8:**
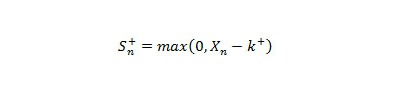
Historical negative binomial cumulative sum (CUSUM) equation. k = reference value, S_n_ = CUSUM score, X_n_ = the case count of the current week, + superscript refers to values always being positive.

Historical NBC is a seasonally adjusted negative binomial CUSUM [20,21]. It calculates *c_0_*, *r*, and *k^+^*using equations in Figure 7 (parts a, b, and e, respectively). The baseline data are patient case counts of the current period during the past 5 years. The calculation of mean (X-bar) and variance (sigma^2^) is based on the given baseline period of the past years. The CUSUM score is given by equation shown in Figure 8.

An outbreak is declared if S_n_^+ ^> *h*, where *h *is the maximum limit for the results to remain in a tolerable state. We used *h *= 15 [18,22] for our analysis. As an outbreak can exist on the very first calculation, generally a shorter period of 5 years is used as a baseline [18,19,22].

### Performance Metrics

To understand how Google Flu Trends data can be used to build an early epidemic detection system, we compared the results of 24 variants of 8 base algorithms (from three categories of algorithms) across three regions in the United States. To the best of our knowledge, this paper presents the first comparative analysis of epidemic detection algorithms for Google Flu Trends data.

For our base algorithms, we used EARS CUSUM, HCusum, HLM, POD, SaTScan, PSC, NBC, and HNBC. The characteristics of these algorithms afford a degree of diversity in our analysis: EARS CUSUM and NBC were designed for rapid detection of outbreaks; HCusum, HNBC, HLM, and POD incorporate seasonal changes but require a substantial training period; and SaTScan requires minimal training and offers flexibility in detecting statistically significant disease clusters.

**Figure 9:**
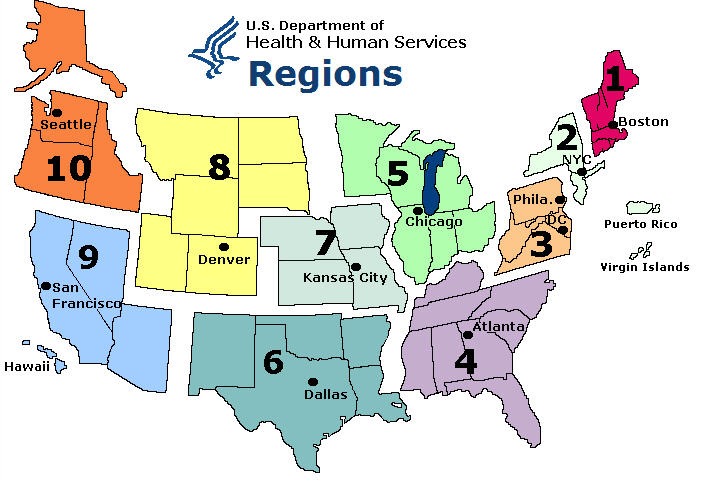
US Department of Health and Human Services regions.

We chose the target regions, as divided by the CDC, to compare the sensitivity of the various algorithms to population size and Internet penetration. Table 1 shows the population size and Internet penetration across HHS regions used in our experiments. Figure 9 maps the regions to US states. We calculated the population of each region by adding the populations of the states in that region. The population in 2009 was used for this calculation [25]. Internet use was taken from a report of the Current Population Survey of Internet Use 2009, published by the National Telecommunications and Information Administration [26] and in the 2009 census published by the US Census Bureau [27].

For our comparison with respect to population size, we focused on region 4 (with the largest population) and region 10 (smallest population). For evaluating the impact of Internet penetration, we focused on region 6 (lowest Internet penetration) and region 10 (highest Internet penetration). Results from region 10 are of particular interest, since it has the lowest population and highest Internet penetration. We expect that the results from region 10 could serve as a benchmark of how accurately Google Flu Trends data can be used as a basis for detecting epidemics. Furthermore, the weather in regions 4 and 6 was similar but very different from that in region 10.

In our analysis, we evaluated each algorithm by comparing its results using Google Flu Trends data with the disease cases reported by CDC. We compared the performance of the algorithms on the following key metrics.

#### Percentage True-Positives

Percentage true-positives (RTP) measures the percentage of time an epidemic signaled in the CDC data is also detected by the target algorithm on Google Flu Trends data. This percentage is calculated by the number of outbreak intervals when the signal was raised divided by the total number of outbreak intervals, with the result multiplied by 100.

#### Percentage False-Positives

Percentage false-positives (RFP) measures the percentage of time an epidemic not signaled in the CDC data is detected as an epidemic by the target algorithm on Google Flu Trends data. This percentage is calculated by the number of nonoutbreak weeks when a signal was raised divided by the total number of weeks with no outbreak, with the result multiplied by 100.

#### Percentage Overlap Time

Percentage overlap (OT) measures the percentage of the time an epidemic detected by an algorithm overlaps with the epidemic signaled in CDC data. Any part of a signal that does not overlap with the original outbreak is not considered in OT.

#### Percentage Early Alarms

Percentage early alarms (EA) measures the percentage of time an algorithm raises an alarm on Google Flu Trends before it is signaled as an epidemic by the CDC data. The early alarm period is limited to the 2 weeks before the start of the original outbreak. Part of a signal starting before this 2-week time period is considered false-positive.

These four metrics capture different aspects of the detection algorithms. RTP measures the sensitivity of an algorithm to outbreaks. At the same time, an overly sensitive algorithm generates a higher number of RFPs.

The average overlap time captures the stability of an algorithm to transient changes in the rate of disease cases. Algorithms that signal the entire period of an epidemic are more desirable than those that raise short, sporadic signals.

Finally, algorithms that signal an epidemic ahead of other algorithms are more suited for early epidemic detection. However, this metric must be viewed in conjunction with an algorithm’s RFP to discount algorithms that generate spurious signals. For our analysis, we counted a signal as an early alarm if its fell within a 2-week window preceding the signal in the CDC data, so long as it was not a continuation of a previous alarm.

**Table 1:** Population and percentage Internet use by US Department of Health and Human Services (HHS) region.

HHS region	Population (2009 census)	% Internet use	States
1	14,412,684	74.07	CT, ME, MA, NH, RI, VT
2	28,224,114	70.20	NJ, NY
3	29,479,361	69.30	DE, DC, MD, PA, VA, WV
4	60,088,178	63.25	AL, FL, GA, KY, MS, NC, SC, TN
5	51,745,410	71.42	IL, IN, MI, MN, OH, WI
6	37,860,549	61.56	AR, LA, NM, OK, TX
7	31,840,178	71.68	IA, KS, MO, NE
8	20,802,785	72.13	CO, MT, ND, SD, UT, WY
9	46,453,010	67.95	AZ, CA, HI, NV
10	6,691,325	76.93	AK, ID, OR, WA

## Results

**Figure 10:**
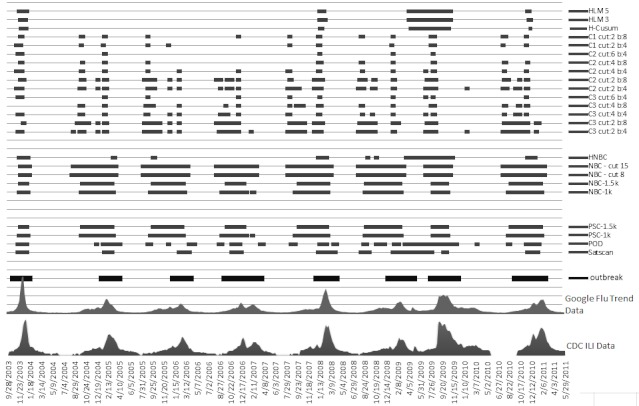
US Department of Health and Human Services region 4. The x-axis plots the Google Flu Trends and Centers for Disease Control and Prevention (CDC) data. The horizontal bars indicate where each method detected an epidemic. Cut indicates the cut-off point (more is less sensitive) and b indicates baseline data (training window). The thick horizontal bars at the bottom show the actual outbreak. HCusum = historical cumulative sum, HLM = historical limits method, HNBC = historical negative binomial cumulative sum, ILI = influenza-like illnesses, k = reference value for threshold, NBC = negative binomial cumulative sum, POD = Poisson outbreak detection, PSC = Poisson cumulative sum.

**Figure 11:**
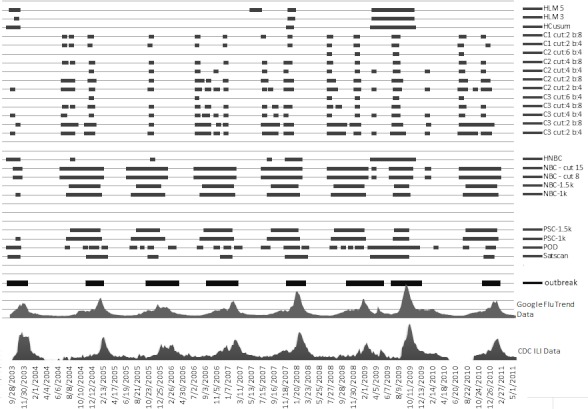
US Department of Health and Human Services region 6. The x-axis plots the Google Flu Trends and Centers for Disease Control and Prevention (CDC) data. The horizontal bars indicate where each method detected an epidemic. Cut indicates cut-off point (more is less sensitive) and b indicates baseline data (training window). The thick horizontal bar at the bottom shows the actual outbreak. HCusum = historical cumulative sum, HLM = historical limits method, HNBC = historical negative binomial cumulative sum, ILI = influenza-like illnesses, k = reference value for threshold, NBC = negative binomial cumulative sum, POD = Poisson outbreak detection, PSC = Poisson cumulative sum.

**Figure 12:**
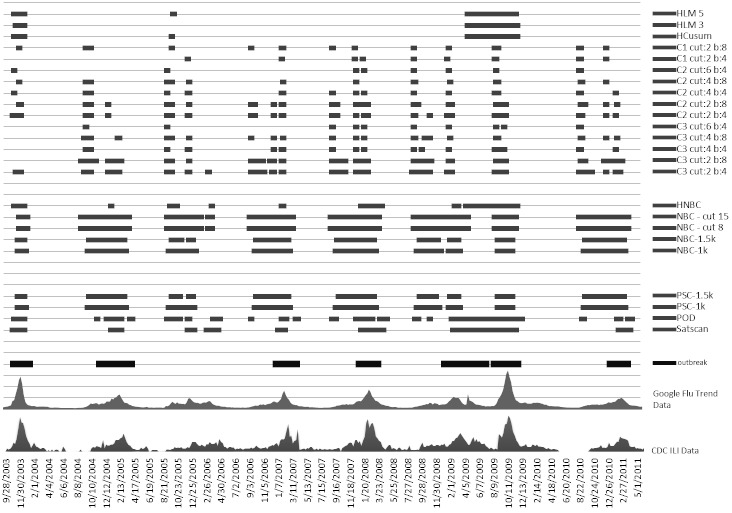
US Department of Health and Human Services region 10. The x-axis plots the Google Flu Trends and Centers for Disease Control and Prevention (CDC) data. The horizontal bars indicate where each method detected an epidemic. Cut indicates cut-off point (more is less sensitive) and b indicates baseline data (training window). The thick horizontal bar at the bottom shows actual outbreak. HCusum = historical cumulative sum, HLM = historical limits method, HNBC = historical negative binomial cumulative sum, ILI = influenza-like illnesses, k = reference value for threshold, NBC = negative binomial cumulative sum, POD = Poisson outbreak detection, PSC = Poisson cumulative sum.

Figure 10, Figure 11, and Figure 12 compare all of the algorithms in our study on a 9-year timescale between 2003 and 2011. Details for these figures are presented in Multimedia Appendix 1, Multimedia Appendix 2, and Multimedia Appendix 3, which compare the algorithms according to our four comparison metrics: RTP, RFP, OT, and EA across our three target regions [12,13,22].

In each Multimedia Appendix there is a sorted column (overall position of algorithm). In this column the algorithms are sorted based on their median across the four performance metrics. We chose the median to cater for extreme values in the performance metrics.

Although we have divided the algorithms into three categories, namely Poisson, negative binomial, and normal distribution algorithms, another subcategory called historical algorithms surfaced during our analysis. This is a subset of both negative binomial and normal distribution categories, as it has algorithms in both. HNBC from the negative binomial and HLM, and HCusum from the normal distribution showed a similar pattern of results across the four performance metrics. Therefore, for the remainder of the discussion, we will add the classification of historical algorithm and analyze its results independently.

In Table 2, for the first performance metric, RTP, all the categories had high average values (96.4%, 99.0%, and 98.8% for normal, NBC, and Poisson distribution algorithms, respectively), with the only exception being the historical algorithms (64%). Moreover, among the algorithms showing a high RTP percentage, there were no significant differences between the values.

In the second performance metric, RFP, values go the other way round, with the historical algorithms showing remarkably optimal values (average 3.3%, where lower is better), whereas normal, NBC, and Poisson distribution algorithms show percentages of 11.4%, 28.3%, and 17.5%, respectively. Clearly the historical algorithms and normal distribution algorithms led in this metric.

In the third metric, OT, negative binomial distribution algorithms led, with an OT of 71.3%, followed by Poisson distribution (60.3%), historical algorithms (30.8%), and normal distribution algorithms (16.4%). In this metric, NBC and Poisson distribution led by a major difference, ahead of historical and normal distribution algorithms.

In the fourth and last metric, EA, negative binomial, on average, led with an EA value of 75.8%, followed by Poisson distribution (55.1%), normal distribution (36.8%) and historical algorithms (22.3%).

For some performance metrics, certain categories did not perform consistently, and values of these categories varied over a large range. In normal distribution algorithms, the values of EA varied from 0% to 75%. In Poisson distribution algorithms, EA varied from 13% to 75%. Therefore, in these cases the average value of that particular metric could not be considered representative, and we needed to examine the algorithms (or variations of algorithms) for suitability.

When we looked at EA values in normal distribution algorithms, the C3 variations of EARS showed a high EA value for only one region. Otherwise, the next best values were barely in the optimal range. Moreover, the OT of C3 at best was 34, which is very low and made this algorithm not suitable.

In case of the EA values in Poisson distributions, the SaTScan algorithm pulled the average of Poisson distribution algorithms down in EA. Therefore, if we considered the average EA value of Poisson distribution algorithms without SaTScan, it actually rose from 55.1 to 66.7.

**Figure 13:**
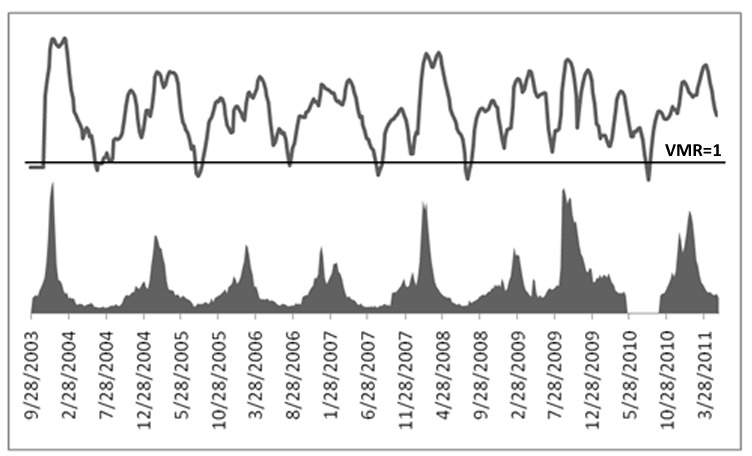
US Centers for Disease Control and Prevention data with the variance to mean ratio (VMR) line above, along the VMR = 1 mark.

Overall, negative binomial and Poisson distribution algorithms performed much better than normal distribution algorithms. This is mainly because of the data distribution that these algorithms expect. The VMR of seasonal influenza-like illness data was greater than 1, most of the time (Figure 13). Therefore, the data followed a negative binomial distribution [28]. Moreover, the Poisson distribution was an approximation of the negative binomial distribution [29,30]. Therefore, the overall percentages of both Poisson-based and negative binomial-based algorithms turned out to be high.

Historical algorithms performed poorly because they considered data during the same period in past years to declare the outbreak. They did not consider the distribution of data during the current year. This made them robust in terms of false-positives, but the performance across other metrics lagged by a substantial difference.

Furthermore, to understand the impact of population variation and change in Internet penetration across regions, we picked the top two algorithms from the negative binomial distribution and Poisson distribution algorithms and applied them to all the regions (instead of just three). Table 3, Table 4, Table 5, and Table 6 present the results of the algorithms applied.

The result of this analysis showed that in regions of high Internet penetration the RFP and OT were high.

**Table 2:** Average percentages of various performance metrics for various categories of algorithms.

Metric	Normal	Negative binomial	Poisson	Historical
RTP^a^	96.4	99.0	98.8	64.0
RFP^b^	11.4	28.3	17.5	3.3
OT^c^	16.4	71.3	60.3	30.8
EA^d^	36.8	75.8	55.1	22.3

^a ^Percentage true-positives.

^b ^Percentage false-positives.

^c ^Percentage overlap time.

^d ^Percentage early alarms.

**Table 3:** Result of negative binomial cumulative sum (cut-off = 15), for all performance metrics across all Department of Health and Human Services (HHS) regions of the United States.

HHS region	RTP^a^	RFP^b^	OT^c^	EA^d^
1	100	45	98	87.5
2	100	40	85	77.7
3	100	40	88	87.5
4	100	30	81	88
5	100	40	95	87.5
6	100	40	76	88
7	100	40	95	87.5
8	87.5	50	83	75
9	90	40	71	80
10	100	40	82	71

^a ^Percentage true-positives.

^b ^Percentage false-positives.

^c ^Percentage overlap time.

^d ^Percentage early alarms.

**Table 4:** Result of negative binomial cumulative sum (threshold = 1 * k) for all performance metrics across all Department of Health and Human Services (HHS) regions of the United States.

HHS region	RTP^a^	RFP^b^	OT^c^	EA^d^
1	100	35	87	87.5
2	100	27	74	66.7
3	100	20	81	75
4	100	20	70	75
5	100	30	86	75
6	100	20	63	75
7	100	30	87	75
8	87.5	40	71	75
9	90	30	64	70
10	100	30	68	71

^a ^Percentage true-positives.

^b ^Percentage false-positives.

^c ^Percentage overlap time.

^d ^Percentage early alarms.

**Table 5:** Result of Poisson cumulative sum (threshold = 1 * k) for all performance metrics across all Department of Health and Human Services (HHS) regions of the United States.

HHS region	RTP^a^	RFP^b^	OT^c^	EA^d^
1	100	35	83	87.5
2	100	27	71	66.7
3	100	20	80	75
4	100	20	70	75
5	100	30	84	75
6	100	20	62	75
7	100	30	84	75
8	87.5	40	67	75
9	90	30	64	70
10	100	30	68	57

^a ^Percentage true-positives.

^b ^Percentage false-positives.

^c ^Percentage overlap time.

^d ^Percentage early alarms.

**Table 6:** Result of Poisson outbreak detection for all performance metrics across all Department of Health and Human Services (HHS) regions of the United States.

HHS region	RTP^a^	RFP^b^	OT^c^	EA^d^
1	100	35	77	33
2	100	20	70	40
3	100	30	69	50
4	100	20	58	75
5	100	40	72	50
6	100	20	50	75
7	100	30	72	75
8	87.5	30	74	75
9	90	20	57	40
10	100	20	68	57

^a ^Percentage true-positives.

^b ^Percentage false-positives.

^c ^Percentage overlap time.

^d ^Percentage early alarms.

## Discussion

In this study, we augmented the capabilities of Google Flu Trends by evaluating various algorithms to translate the raw search query volume produced by this service into actionable alerts. We focused, in particular, on leveraging the ability of Google Flu Trends to provide a near real-time alternative to conventional disease surveillance networks and to explore the practicality of building an early epidemic detection system using these data. This paper presents the first detailed comparative analysis of popular early epidemic detection algorithms on Google Flu Trends. We explored the relative merits of these methods and considered the effects of changing Internet prevalence and population sizes on the ability of these methods to predict epidemics. In these evaluations, we drew upon data collected by the CDC and assessed the ability of each algorithm within a consistent experimental framework to predict changes in measured CDC case frequencies from the Internet search query volume.

Our analysis showed that adding a layer of computational intelligence to Google Flu Trends data provides the opportunity for a reliable early epidemic detection system that can predict disease outbreaks with high accuracy in advance of the existing systems used by the CDC. However, we note that realizing this opportunity requires moving beyond the CUSUM- and HLM-based normal distribution approaches traditionally employed by the CDC. In particular, while we did not find a single best method to apply to Google Flu Trends data, the results of our study strongly support negative binomial- and Poisson-based algorithms being more useful when dealing with potentially noisy search query data from regions with varying Internet penetrations. For such data, we found that normal distribution algorithms did not perform as well as the negative binomial and Poisson distribution algorithms.

Furthermore, our analysis showed that the patient data of a disease follows different distributions throughout the year. Therefore, when VMR of data is equal to 1, it is ideally following a Poisson distribution and could be handled by a Poisson-based algorithm. As the increase in variance raises VMR above 1, the data become overdispersed. Poisson-based algorithms can handle this overdispersion, up to a limit [29]. When VMR is very high, an algorithm is needed that considers the variance as a parameter and raises alarms accordingly. Since negative binomial distribution-based algorithms consider the variance [29], such algorithms perform better in similar scenarios. For instance, NBC is accurate in raising an alarm for overdispersed count data [29]. To get better results, we propose an approach, based on the above discussion, of changing the distribution expectation of an algorithm along with the rise and fall in VMR. This area should be explored in more depth to produce algorithms that adapt according to the data’s distribution type.

Our research is the first attempt of its kind to relate epidemic prediction using Google Flu Trends data to Internet penetration and the size of the population being assessed. We believe that understanding how these factors affect algorithms to predict epidemics is an integral question for scaling a search query-based system to a broad range of geographical regions and communities. In our investigations, we observed that both Internet penetration and population size had a definite impact on algorithm performance. SaTScan performs better when applied to data from regions with high Internet penetration and small population size, while POD and NBC achieves better results when Internet penetration is low and population size is large. CUSUM performs best in regions with a large population. While the availability of search query data and measured (ie, CDC) case records restrict our analyses to the United States, we believe many of these insights may be useful in developing an early epidemic prediction system for other regions, including communities in the developing world.

In conclusion, we present an early investigation of algorithms to translate data from services such as Google Flu Trends into a fully automated system for generating alerts when the likelihood of epidemics is quite high. Our research augments the ability to detect disease outbreaks at early stages, when many of the conditions that impose an immense burden globally can be treated with better outcomes and in a more cost-effective manner. In addition, the ability to respond early to imminent conditions allows for more proactive restriction of the size of any potential outbreak. Together, the findings of our study provide a means to convert raw data collected over the Internet into more fine-grained information that can guide effective policy in countering the spread of diseases.

Based on our work, we have developed FluBreaks (dritte.org/flubreaks), an early warning system for flu epidemics using Google Flu Trends.

## Multimedia Appendix 1

Ranking of algorithms in different parameters of evaluation for HSS Region 4 (Highest Population).

## Multimedia Appendix 2

Ranking of algorithms in different parameters of evaluation for HSS Region 6 (Lowest Percent Internet Use).

## Multimedia Appendix 3

Ranking of algorithms in different parameters of evaluation for HSS Region 10 (Lowest Population and Highest Percent Internet Use).

## Abbreviations

CDC: Centers for Disease Control and Prevention
CUSUM: cumulative sum
EA: percentage early alarms
EARS: Early Aberration Reporting System
HCusum: historical cumulative sum
HLM: historical limits method
ILINet: Outpatient Influenza-like Illness Surveillance Network
NBC: negative binomial cumulative sum
OT: percentage overlap
POD: Poisson outbreak detection
PSC: Poisson cumulative sum
RFP: percentages false-positives
RTP: percentage true-positives
VMR: variance to mean ratio
