# Supplementary material for: FluBreaks: Early Epidemic Detection from Google Flu Trends
Source: J Med Internet Res. 2012 Oct 4;14(5):e125. doi: 10.2196/jmir.2102 (PMC3510767; doi:10.2196/jmir.2102)
Supplement: Supplementary file 1 [file jmir_v14i5e125_app1.pdf]

Multimedia Appendix 1: Ranking of algorithms in different parameters of evaluation for HSS Region 4 (Highest Population)

Percentage of True Positive			Percentage of False Positive			Percentage of Overlap			Percentage of Early Alarm			Overall Position of Algorithm		
**Pos**	**Algorithm**	**Value**	**Pos**	**Algorithm**	**Value**	**Pos**	**Algorithm**	**Value**	**Pos**	**Algorithm**	**Value**	**Pos**	**Algorithm**	**Value**
1	C1 - cut:2 b4	100	1	C2 - cut:6 b4	0	1	NBC - cut 8	81	1	NBC - cut 8	88	1	NBC - cut 15	85
1	C2 - cut:2 b4	100	1	HLM 3	0	1	NBC - cut 15	81	1	NBC - cut 15	88	1	NBC - cut 8	85
1	C3 - cut:2 b4	100	1	HLM 5	0	2	NBC - 1k	70	2	C3 - cut:2 b4	75	2	C3 - cut:2 b4	78
1	C2 - cut:4 b4	100	2	H-Cusum	10	2	PSC - 1k	70	2	C3 - cut:2 b8	75	2	C3 - cut:2 b8	78
1	C3 - cut:4 b4	100	2	HNBC	10	3	NBC - 1.5k	60	2	POD	75	2	NBC - 1k	78
1	C2 - cut:6 b4	100	2	NBC - 1.5k	10	3	PSC - 1.5k	60	2	NBC - 1k	75	2	POD	78
1	C3 - cut:6 b4	100	2	PSC - 1.5k	10	4	POD	58	2	PSC - 1k	75	2	PSC - 1k	78
1	C1 - cut:2 b8	100	2	C1 - cut:2 b4	10	5	Satscan	52	3	NBC - 1.5k	63	3	NBC - 1.5k	77
1	C2 - cut:2 b8	100	2	C2 - cut:4 b4	10	6	HNBC	35	3	PSC - 1.5k	63	3	PSC - 1.5k	77
1	C3 - cut:2 b8	100	2	C3 - cut:4 b4	10	7	C3 - cut:2 b8	31	3	C2 - cut:2 b4	63	4	C2 - cut:2 b4	72
1	C2 - cut:4 b8	100	2	C3 - cut:6 b4	10	8	C3 - cut:2 b4	30	3	C2 - cut:2 b8	63	4	C2 - cut:2 b8	72
1	C3 - cut:4 b8	100	2	C1 - cut:2 b8	10	9	C2 - cut:2 b4	27	4	C3 - cut:4 b4	38	5	Satscan	71
1	Satscan	100	2	C2 - cut:4 b8	10	10	H-Cusum	26	4	C1 - cut:2 b8	38	6	C1 - cut:2 b8	64
1	POD	100	2	C3 - cut:4 b8	10	11	HLM 5	24	4	C3 - cut:4 b8	38	6	C3 - cut:4 b4	64
1	NBC - cut 8	100	2	Satscan	10	12	C2 - cut:2 b8	23	5	C1 - cut:2 b4	25	6	C3 - cut:4 b8	64
1	NBC - cut 15	100	3	NBC - 1k	20	12	HLM 3	23	6	HLM 3	20	7	C1 - cut:2 b4	58
1	NBC - 1k	100	3	PSC - 1k	20	13	C3 - cut:4 b4	19	6	HLM 5	20	7	HNBC	58
1	NBC - 1.5k	100	3	C2 - cut:2 b4	20	14	C3 - cut:4 b8	17	7	HNBC	13	8	C2 - cut:6 b4	57
1	PSC - 1k	100	3	C3 - cut:2 b4	20	15	C2 - cut:4 b4	16	7	C2 - cut:4 b4	13	9	C2 - cut:4 b4	53
1	PSC - 1.5k	100	3	C2 - cut:2 b8	20	16	C1 - cut:2 b4	15	7	C2 - cut:6 b4	13	10	C2 - cut:4 b8	52
2	HNBC	80	3	C3 - cut:2 b8	20	17	C1 - cut:2 b8	13	7	C3 - cut:6 b4	13	10	C3 - cut:6 b4	52
3	HLM 3	60	3	POD	20	18	C2 - cut:4 b8	12	7	C2 - cut:4 b8	13	11	H-Cusum	43
3	HLM 5	60	4	NBC - cut 8	30	19	C3 - cut:6 b4	11	7	Satscan	13	12	HLM 5	42
3	H-Cusum	60	4	NBC - cut 15	30	20	C2 - cut:6 b4	9	7	H-Cusum	13	12	HLM 3	42

Note: Value of Percentage of True Positive (RTP) and Percentage of False Positive (RFP) are rounded off to the nearest ten.
