# Supplementary material for: FluBreaks: Early Epidemic Detection from Google Flu Trends
Source: J Med Internet Res. 2012 Oct 4;14(5):e125. doi: 10.2196/jmir.2102 (PMC3510767; doi:10.2196/jmir.2102)
Supplement: Supplementary file 2 [file jmir_v14i5e125_app2.pdf]

Multimedia Appendix 2: Ranking of algorithms in different parameters of evaluation for HSS Region 6 (Lowest Percent Internet Use)

Percentage of True Positive			Percentage of False Positive			Percentage of Overlap			Percentage of Early Alarm			Overall Position of Algorithm		
**Pos**	**Algorithm**	**Value**	**Pos**	**Algorithm**	**Value**	**Pos**	**Algorithm**	**Value**	**Pos**	**Algorithm**	**Value**	**Pos**	**Algorithm**	**Value**
1	C1 - cut:2 b4	100	1	C2 - cut:6 b4	0	1	NBC - cut 8	76	1	NBC - cut 8	88	1	NBC - cut 15	82
1	C2 - cut:2 b4	100	1	HLM 3	0	1	NBC - cut 15	76	1	NBC - cut 15	88	1	NBC - cut 8	82
1	C3 - cut:2 b4	100	1	H-Cusum	0	2	NBC - 1k	63	2	POD	75	2	NBC - 1.5k	78
1	C2 - cut:4 b4	100	1	C3 - cut:6 b4	0	3	PSC - 1k	62	2	NBC - 1k	75	2	NBC - 1k	78
1	C3 - cut:4 b4	100	2	HNBC	10	4	NBC - 1.5k	55	2	PSC - 1k	75	2	POD	78
1	C3 - cut:2 b8	100	2	C2 - cut:4 b4	10	5	PSC - 1.5k	53	2	NBC - 1.5k	75	2	PSC - 1.5k	78
1	Satscan	100	2	Satscan	10	5	Satscan	53	2	PSC - 1.5k	75	2	PSC - 1k	78
1	POD	100	2	HLM 5	10	6	POD	50	3	C3 - cut:2 b4	50	3	Satscan	72
1	NBC - cut 8	100	2	C1 - cut:2 b4	10	7	HNBC	34	4	C2 - cut:2 b8	43	4	C2 - cut:2 b8	65
1	NBC - cut 15	100	2	C1 - cut:2 b8	10	8	H-Cusum	27	4	C3 - cut:4 b8	43	4	C3 - cut:4 b8	65
1	NBC - 1k	100	2	C2 - cut:4 b8	10	9	HLM 5	26	5	C1 - cut:2 b4	38	4	C3 - cut:2 b4	65
1	PSC - 1k	100	2	C3 - cut:4 b4	10	11	HLM 3	22	5	C2 - cut:2 b4	38	5	C1 - cut:2 b4	64
2	NBC - 1.5k	88	2	C3 - cut:4 b8	10	12	C3 - cut:2 b4	19	5	C2 - cut:4 b4	38	5	C2 - cut:2 b4	64
2	PSC - 1.5k	88	2	C2 - cut:2 b8	10	12	C3 - cut:2 b8	19	5	C3 - cut:4 b4	38	5	C2 - cut:4 b4	64
2	C2 - cut:6 b4	88	2	C2 - cut:2 b4	10	13	C2 - cut:2 b4	17	5	C3 - cut:2 b8	38	5	C3 - cut:4 b4	64
2	C3 - cut:6 b4	88	3	NBC - 1.5k	20	13	C2 - cut:2 b8	17	5	HNBC	38	6	C3 - cut:2 b8	59
2	C1 - cut:2 b8	88	3	PSC - 1.5k	20	14	C1 - cut:2 b4	12	5	H-Cusum	38	7	C1 - cut:2 b8	58
2	C2 - cut:2 b8	88	3	C3 - cut:2 b4	20	14	C3 - cut:4 b4	12	6	C1 - cut:2 b8	29	7	C2 - cut:4 b8	58
2	C2 - cut:4 b8	88	3	POD	20	14	C3 - cut:4 b8	12	6	C2 - cut:4 b8	29	8	HNBC	57
2	C3 - cut:4 b8	88	3	C3 - cut:2 b8	20	15	C1 - cut:2 b8	11	7	Satscan	25	9	C3 - cut:6 b4	47
3	HNBC	75	3	NBC - 1k	20	16	C2 - cut:4 b4	10	8	HLM 3	20	9	C2 - cut:6 b4	47
4	HLM 3	63	3	PSC - 1k	20	17	C2 - cut:4 b8	9	8	HLM 5	20	10	HLM 5	44
4	HLM 5	63	4	NBC - cut 8	40	18	C3 - cut:6 b4	7	9	C2 - cut:6 b4	0	10	H-Cusum	44
5	H-Cusum	50	4	NBC - cut 15	40	19	C2 - cut:6b4	6	9	C3 - cut:6 b4	0	11	HLM 3	42

Note: Value of Percentage of True Positive (RTP) and Percentage of False Positive (RFP) are rounded off to the nearest ten.
