# Supplementary material for: FluBreaks: Early Epidemic Detection from Google Flu Trends
Source: J Med Internet Res. 2012 Oct 4;14(5):e125. doi: 10.2196/jmir.2102 (PMC3510767; doi:10.2196/jmir.2102)
Supplement: Supplementary file 3 [file jmir_v14i5e125_app3.pdf]

Multimedia Appendix 3: Ranking of algorithms in different parameters of evaluation for HSS Region 10 (Lowest Population and Highest Percent Internet Use)

Percentage of True Positive			Percentage of False Positive			Percentage of Overlap			Percentage of Early Alarm			Overall Position of Algorithm		
**Pos**	**Algorithm**	**Value**	**Pos**	**Algorithm**	**Value**	**Pos**	**Algorithm**	**Value**	**Pos**	**Algorithm**	**Value**	**Pos**	**Algorithm**	**Value**
1	C1 - cut:2 b4	100	1	H-Cusum	0	1	NBC - cut 8	82	1	NBC - cut 8	71	1	Satscan	77
1	C2 - cut:2 b4	100	1	HNBC	0	1	NBC - cut 15	82	1	NBC - cut 15	71	1	NBC - cut 15	77
1	C3 - cut:2 b4	100	1	HLM 3	0	2	NBC - 1k	68	1	NBC - 1k	71	1	NBC - cut 8	77
1	C2 - cut:4 b4	100	1	HLM 5	0	2	PSC - 1k	68	2	POD	57	2	POD	74
1	C3 - cut:4 b4	100	1	C2 - cut:6 b4	0	2	Satscan	68	2	C3 - cut:2 b4	57	3	NBC - 1.5k	71
1	C1 - cut:2 b8	100	2	C1 - cut:2 b4	10	2	POD	68	2	C3 - cut:2 b8	57	3	PSC - 1.5k	71
1	C2 - cut:2 b8	100	2	C2 - cut:4 b4	10	3	NBC - 1.5k	62	2	PSC - 1k	57	3	NBC - 1k	71
1	C3 - cut:4 b8	100	2	C3 - cut:4 b4	10	3	PSC - 1.5k	62	2	NBC - 1.5k	57	4	HNBC	70
1	C3 - cut:2 b8	100	2	C3 - cut:6 b4	10	4	HNBC	54	2	PSC - 1.5k	57	5	PSC - 1k	69
1	POD	100	2	C1 - cut:2 b8	10	5	H-Cusum	35	3	C1 - cut:2 b4	43	5	C3 - cut:2 b4	69
1	NBC - cut 8	100	2	C2 - cut:2 b8	10	6	C3 - cut:2 b4	34	3	C2 - cut:2 b4	43	5	C3 - cut:2 b8	69
1	NBC - cut 15	100	2	C2 - cut:4 b8	10	7	C3 - cut:2 b8	33	3	C3 - cut:4 b4	43	6	C1 - cut:2 b4	67
1	NBC - 1k	100	2	C3 - cut:4 b8	10	8	HLM 3	32	3	C1 - cut:2 b8	43	6	C1 - cut:2 b8	67
1	PSC - 1k	100	2	Satscan	10	8	HLM 5	32	3	C2 - cut:2 b8	43	6	C2 - cut:2 b8	67
1	NBC - 1.5k	100	3	PSC - 1.5k	20	10	C2 - cut:2 b4	28	3	C3 - cut:4 b8	43	6	C3 - cut:4 b4	67
1	PSC - 1.5k	100	3	NBC - 1.5k	20	11	C2 - cut:2 b8	25	4	H-Cusum	29	6	C3 - cut:4 b8	67
2	HNBC	86	3	C2 - cut:2 b4	20	12	C3 - cut:4 b4	18	4	C2 - cut:4 b4	29	7	C2 - cut:2 b4	62
2	C3 - cut:6 b4	86	3	C3 - cut:2 b4	20	13	C2 - cut:4 b4	16	4	C3 - cut:6 b4	29	8	C2 - cut:4 b4	60
2	C2 - cut:4 b8	86	3	C3 - cut:2 b8	20	14	C1 - cut:2 b4	14	4	C2 - cut:4 b8	29	9	C2 - cut:4 b8	57
2	Satscan	86	3	POD	20	14	C3 - cut:4 b8	14	4	HLM 3	29	9	C3 - cut:6 b4	57
3	H-Cusum	71	4	NBC - 1k	30	15	C1 - cut:2 b8	12	5	HNBC	14	10	H-Cusum	53
3	C2 - cut:6 b4	71	4	PSC - 1k	30	16	C3 - cut:6 b4	10	5	C2 - cut:6 b4	14	11	HLM 5	45
4	HLM 5	57	5	NBC - cut 8	40	17	C2 - cut:4 b4	9	5	Satscan	14	12	C2 - cut:6 b4	43
5	HLM 3	43	5	NBC - cut 15	40	17	C2 - cut:6 b4	9	5	HLM 5	14	13	HLM 3	37

Note: Value of Percentage of True Positive (RTP) and Percentage of False Positive (RFP) are rounded off to the nearest ten.
